# Supplementary material for: The position of single-base deletions in the VNTR sequence of the carboxyl ester lipase (CEL) gene determines proteotoxicity
Source: J Biol Chem. 2021 Apr 14;296:100661. doi: 10.1016/j.jbc.2021.100661 (PMC8692231; doi:10.1016/j.jbc.2021.100661)
Supplement: Figures S1 and S2 [file mmc1.pdf]

Supporting information

The position of single-base deletions in the VNTR sequence of the carboxyl ester lipase (*CEL*) gene determines proteotoxicity

Anny Gravdal[a,b,c], Xunjun Xiao[d], Miriam Cnop[e,f], Khadija El Jellas[a,b], Stefan Johansson[b,c], Pål R. Njølstad[b,g], Mark E. Lowe[d], Bente B. Johansson[b,g], Anders Molven[a,b,h], Karianne Fjeld[a,b,c]

[a] The Gade Laboratory for Pathology, Department of Clinical Medicine, University of Bergen, Bergen, Norway
[b] Center for Diabetes Research, Department of Clinical Science, University of Bergen, Norway
[c] Department of Medical Genetics, Haukeland University Hospital, Bergen, Norway
[d] Department of Pediatrics, Division of Gastroenterology, Hepatology and Nutrition, Washington University School of Medicine, St. Louis, MO, USA
[e] ULB Center for Diabetes Research, Université Libre de Bruxelles, Brussels, Belgium
[f] Division of Endocrinology, ULB Erasmus Hospital, Université Libre de Bruxelles, Brussels, Belgium
[g] Department of Pediatrics and Adolescent Medicine, Haukeland University Hospital, Bergen, Norway
[h] Department of Pathology, Haukeland University Hospital, Bergen, Norway

CONTENTS

Figure S1. Potential O-glycosylation sites of the VNTR region of the normal CEL protein (WT) and of the investigated DEL variants.

Figure S2. Predicted amino acid sequence of the VNTR region of the normal CEL protein (WT) and of the investigated DEL variants.

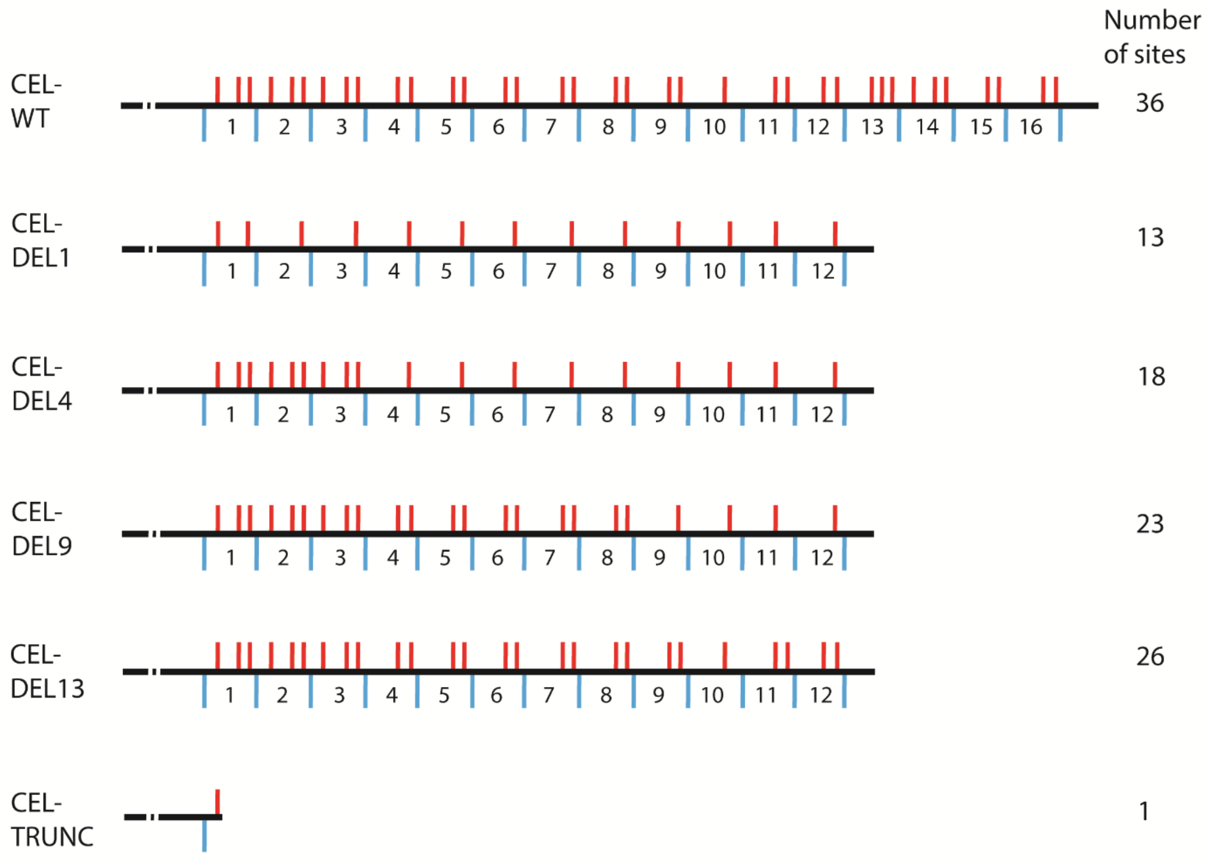

Figure S1. Potential O-glycosylation sites of the VNTR region of the normal CEL protein (WT) and of the investigated DEL variants. For each CEL variant, theoretical O-glycosylation sites were predicted by the NetOGlyc3.1 server at http://www.cbs.dtu.dk/services/NetOGlyc. These sites were all located in the VNTR region and are indicated as red bars above the black line, with the total number of predicted sites listed on the right. VNTR segment numbers and borders (blue lines) of each variant are shown below the black line. For all deletion variants, the reading frame terminates within repeat 13. As indicated, the truncated control variant of CEL (TRUNC) has only one predicted O-glycosylation site.

	1	2	3	4
**WT**	EATPVPPTGDS	EATPVPPTGDS	ETAPVPPTGDS	GAPPVPPTGDS
**DEL1**	EATPCPPQGTP	RPLPCPPRVTP	RPPPCRPRVTP	GPPPCRPRVTP
**DEL4**	EATPVPPTGDS	EATPVPPTGDS	ETAPVPPTGDS	GAPPCRPRVTP
**DEL9**	EATPVPPTGDS	EATPVPPTGDS	ETAPVPPTGDS	GAPPVPPTGDS
**DEL13**	EATPVPPTGDS	EATPVPPTGDS	ETAPVPPTGDS	GAPPVPPTGDS
**TRUNC**	EAT			

	5	6	7	8
**WT**	GAPPVPPTGDS	GAPPVPPTGDS	GAPPVPPTGDS	GAPPVPPTGDS
**DEL1**	GPPPCRPRVTP	GPPPCRPRVTP	GPPPCRPRVTP	GPPPCRPRVTP
**DEL4**	GPPPCRPRVTP	GPPPCRPRVTP	GPPPCRPRVTP	GPPPCRPRVTP
**DEL9**	GAPPVPPTGDS	GAPPVPPTGDS	GAPPVPPTGDS	GAPPVPPTGDS
**DEL13**	GAPPVPPTGDS	GAPPVPPTGDS	GAPPVPPTGDS	GAPPVPPTGDS

	9	10	11	12
**WT**	GAPPVPPTGDS	GAPPVPPTGDA	GPPPVPPTGDS	GAPPVPPTGDS
**DEL1**	GPPPCRPRVTP	APPPCRPRVTP	GPPPCRPRVTP	APPPCRPRVTP
**DEL4**	GPPPCRPRVTP	APPPCRPRVTP	GPPPCRPRVTP	APPPCRPRVTP
**DEL9**	GAPPCRPRVTP	APPPCRPRVTP	GPPPCRPRVTP	APPPCRPRVTP
**DEL13**	GAPPVPPTGDS	GAPPVPPTGDA	GPPPVPPTGDS	GAPPVPPTGDS

	13	14	15	16
**WT**	GAPPVTPTGDS	ETAPVPPTGDS	GAPPVPPTGDS	EAAPVPPTDDS
**DEL1**	GPPP			
**DEL4**	GPPP			
**DEL9**	GPPP			
**DEL13**	GAPP			

Figure S2. Predicted amino acid sequence of the VNTR region of the normal CEL protein (WT) and of the investigated DEL variants. One-letter amino acid nomenclature is used, with predicted glycosylated residues (serine, S; threonine, T) highlighted in red. Cysteine residues (C), which may potentially form intra- and intermolecular disulfide bridges, are highlighted in black/green. The truncated control variant of CEL (TRUNC) terminates after the three first amino acids in repeat 1.
